# Gut Microbiota-mediated Alleviation of Dextran Sulfate Sodium-induced Colitis in Mice

**DOI:** 10.1016/j.gastha.2024.01.016

**Published:** 2024-02-06

**Authors:** Eri Ikeda, Masaya Yamaguchi, Shigetada Kawabata

**Affiliations:** 1 Department of Microbiology, Graduates School of Dentistry, Osaka University, Suita, Osaka, Japan; 2 Department of Molecular Immunology, Graduate School of Medical and Dental Sciences, Tokyo Medical and Dental University, Tokyo, Japan; 3 Bioinformatics Research Unit, Graduates School of Dentistry, Osaka University, Suita, Osaka, Japan; 4 Center for Infectious Disease Education and Research (CiDER), Osaka University, Suita, Osaka, Japan; 5 Bioinformatics Center, Research Institute for Microbial Diseases, Osaka University, Suita, Osaka, Japan

**Keywords:** butyrate, IBD, DSS, Roseburia, SCFA

## Abstract

**Background and Aims:**

Gut dysbiosis characterized by an imbalanced microbiota is closely involved in the pathogenesis of a widespread gastrointestinal inflammatory disorder, inflammatory bowel disease. However, it is unclear how the complex intestinal microbiota affects development or resistant of mucosal inflammation. Our aim was to investigate the impact of the gut microbiota on susceptibility in a mouse model of ulcerative colitis.

**Methods:**

We compared the susceptibility to dextran sulfate sodium (DSS)-induced colitis of inbred BALB/c mice obtained from the 3 main distributors of laboratory animals in Japan. Clinical symptoms of the colitis and the faecal microbiota were assessed. Cohousing approach was used to identify whether the gut microbiota is a primary factor determining disease susceptibility.

**Results:**

Here, we showed differences in the susceptibility of BALB/c mice from the vendors to DSS colitis. Analysis of the gut microbiota using 16S ribosomal RNA sequencing revealed clear separation of the gut microbial composition among mice from the vendors. Notably, the abundance of the phylum *Actinobacteriota* was strongly associated with disease activity. We also observed the expansion of butyrate-producing *Roseburia* species in mice with decreased susceptibility of the disease. Further cohousing experiments showed that variation in clinical outcomes was more correlated with the gut microbiota than genetic variants among substrains from different suppliers.

**Conclusion:**

A BALB/c substrain that was resistant to DSS-induced colitis was observed, and the severity of DSS-induced colitis was mainly influenced by the gut microbiota. Targeting butyrate-producing bacteria could have therapeutic potential for ulcerative colitis.

## Graphical abstract

**Figure:**
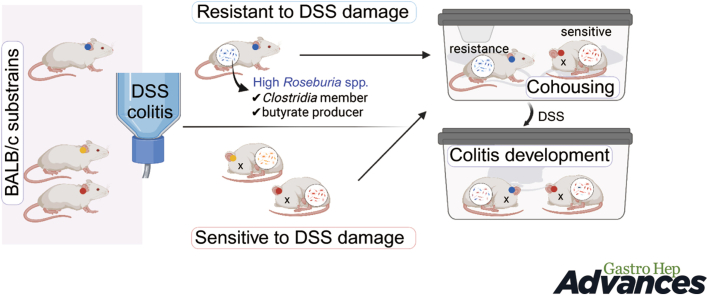

## Introduction

Laboratory mice are important species for preclinical animal experiments in biomedical research and contribute to mechanistic studies and drug development in the context of various human diseases. Inbred mouse strains difference can be a reason of host immune characteristics as well as behavioural phenotypes.^1^^,^^2^ Numerous substrains have been derived from original inbred strains.^3^ Substrains are defined as branches of an inbred strain produced by separated brother-sister mating over at least 20 generations from multiple vendors. During inbreeding, substrains from the classic inbred line diverge over time; thus, although these substrains are related, they are not identical because of housing conditions.^4^, ^5^, ^6^

The gastrointestinal tract of mammals is considered a primary reservoir for microorganisms that comprise vast and complex communities.^7^, ^8^, ^9^ The gut microbiota, which includes hundreds of bacterial species, has been extensively investigated through epidemiological, physiological, and omics-based studies in humans.^10^, ^11^, ^12^ Accumulating evidence suggests that disruption of the gut microbiome, which is known as dysbiosis, is closely connected to pathological states such as inflammation, autoimmune disorders, and cancer.^13^, ^14^, ^15^ Inflammatory bowel disease (IBD) is a multifactorial immune-mediated inflammatory disease that includes 2 conditions, Crohn’s disease and ulcerative colitis.^16^^,^^17^ The gut dysbiosis associated with IBD is characterized by a lack of diversity, an increase in the levels of *Proteobacteria*, such as *Enterobacteriaceae* and *BIlophila*, and reduced levels of beneficial bacteria, such as short-chain fatty acid (SCFA)-producing *Clostridium*.^18^, ^19^, ^20^, ^21^ SCFAs produced by gut bacteria act as coenzymes in fat and carbohydrate metabolism, thus exhibiting anti-inflammatory effects in IBD. Among the SCFAs, butyrate serves as a principal energy source for intestinal wound healing and barrier function. The gut microbiota of IBD patients exhibits a selective decrease in the levels of butyrate producers, and the colonocytes of IBD patients are incapable of transferring and utilizing butyrate.^22^

The dextran sulfate sodium (DSS)-induced colitis model is routinely used as a principal mouse model of ulcerative colitis. DSS administration in drinking water mediates gut epithelial damage, which causes inflammation. Mouse strain and sex differences influence colitis susceptibility.^23^ For example, similar to the clinical development of ulcerative colitis in humans, male mice are more likely to develop DSS-induced colitis than female mice.^24^^,^^25^ Additionally, BALB/c mice require higher concentrations of DSS to induce colitis than C57BL/6J mice.^24^ C57BL/6 wild-type mice from the same inbred strain purchased from 2 vendors (substrains), Jackson laboratory and Taconic farms, showed different bacterial compositions, and Jackson mice showed significantly fewer species of bacteria.^26^ Colonization of the gut by a segmented filamentous bacterium was found only in Taconic mice, and the bacterium induced the production of inflammatory Th17 cells in the lamina propria of the small intestine, which resulted in increased resistance to *Citrobacter rodentium*-induced colitis.^26^ In addition, the composition of gut microbes that influence susceptibility to several diseases, including abdominal sepsis, vary among substrains.^27^

Despite these advances in understanding, whether the gut microbiota is a primary factor determining disease susceptibility in DSS-induced colitis remains unknown. We compared mice from the same inbred strain (BALB/c) that were obtained from the 3 main distributors of laboratory animals in Japan and identified considerable variability in the presentation of DSS-induced colitis among mice from different vendors. We, therefore, quantified the influence of the faecal microbiota associated with mice from different vendors. We identified that mice from each vendor harboured a distinct gut microbiota. Thus, using a cohousing approach, we revealed that disease resistance mainly relies on the gut microbiota and not on genetic differences among substrains.

## Methods

### Animals and DSS-Induced Colitis Model

Female BALB/C wild-type mice were obtained from 3 animal vendors: SLC Japan (BALB/cCrSLC, Shizuoka, Japan), CLEA Japan (BALB/cAJcl, Tokyo, Japan), and Charles River Laboratories Japan (BALB/cAnNCrlCrlj Kanagawa, Japan). Mice were randomly assigned to cages before the experiment. Six-to seven-week-old mice were used except in the cohousing experiment. Acute colitis was induced by orally administrating 4% (w/v) DSS (molecular mass 36–50 kDa; MP biomedicals) in the drinking water for 8 days. Body weight loss (0, none; 1, 1%–5%; 2, 5%–10%; 3, 10%–20%; 4, > 20%), rectal bleeding (0, normal; 2, haemoccult positive; 4, gross blood), and stool consistency (0, normal stool; 2, loose stool; 4, diarrhoea) were monitored daily. These parameters were used to assess the colitis clinical score and the disease activity index (DAI). Mice were euthanized on day 8. Colon length was measured as the distance between the end of the caecum and proximal rectum.

All animal experiments were approved by the Institutional Animal Care and Use Committee of Tokyo Medical and Dental University (Protocol number: A2021-088C9) and the Animal Care and Use Committee of Osaka University Graduate School of Dentistry (R05-009-0). All animals that were used in this study were housed in groups of 3–6 mice, fed standard pellet diet, under a 12-hour light/dark cycle.

### 16S rRNA Sequencing

Mouse faeces or colon luminal contents were collected on day 0 and day 8. Collected samples were stored at −80 °C until further use. Bacterial DNA was isolated using a NucleoSpin DNA stool kit (Takara Bio, Shiga, Japan). The V3-V4 regions of the 16S ribosomal RNA (rRNA) gene were amplified in each sample. Sequencing was performed on the Illumina MiSeq platform using a MiSeq Reagent Kit V3 (300 bp x 2) (Eurofins genomics, Tokyo, Japan and Bioengineering Lab. Co., Ltd., Kanagawa, Japan).

Raw sequences were curated using the software package Qiime2. Sequences were assigned to operational taxonomic units using a cut-off = 0.03 and classified using the SILVA platform with a 70% confidence threshold. We used the linear discriminant analysis effect size (LEfSe) method^28^ (http://huttenhower.sph.harvard.edu/lefse), which is used to perform a combined assessment of statistical significance and biological relevance.

### Cohousing

For the cohousing experiment, four-week-old BALB/C female mice purchased from SLC and Charles River were housed separately for one week in the same room and were fed the same diet before cohousing. Then, the mice were transferred into a new cage, and SLC mice and Charles River mice were cohoused for 4 weeks as described previously.^29^ SLC mice or Charles River mice that were kept in separate cages were used as controls. Acute colitis was induced with 4% (w/v) DSS for 7 days afterwards.

### Statistics and Reproducibility

Comparisons of 2 groups were performed using an unpaired *t* (parametric) test or a Mann‒Whitney *U* (nonparametric) test. Differences among more than 3 groups were evaluated using one-way analysis of variance for parametric analysis or the Kruskal‒Wallis test for nonparametric analysis followed by Bonferroni correction (parametric) or Steel-Dwass correction (nonparametric). The normality of the data was analyzed using the Kolmogorov‒Smirnov test. Homogeneity of variance was analyzed using the F test (2 groups) or Bartlett test (more than 3 groups). The sample distribution of the gut microbiota was analyzed by a non-metric multi-dimensional scaling method. Correlation analysis was performed using the Pearson correlation coefficient. Error bars represent the standard deviation of a data set. All statistical analyses were performed with R statistical software.

## Results

### The Phenotype of DSS-Induced Colitis Varied Among Mice From Different Vendors

**Figure 1:**
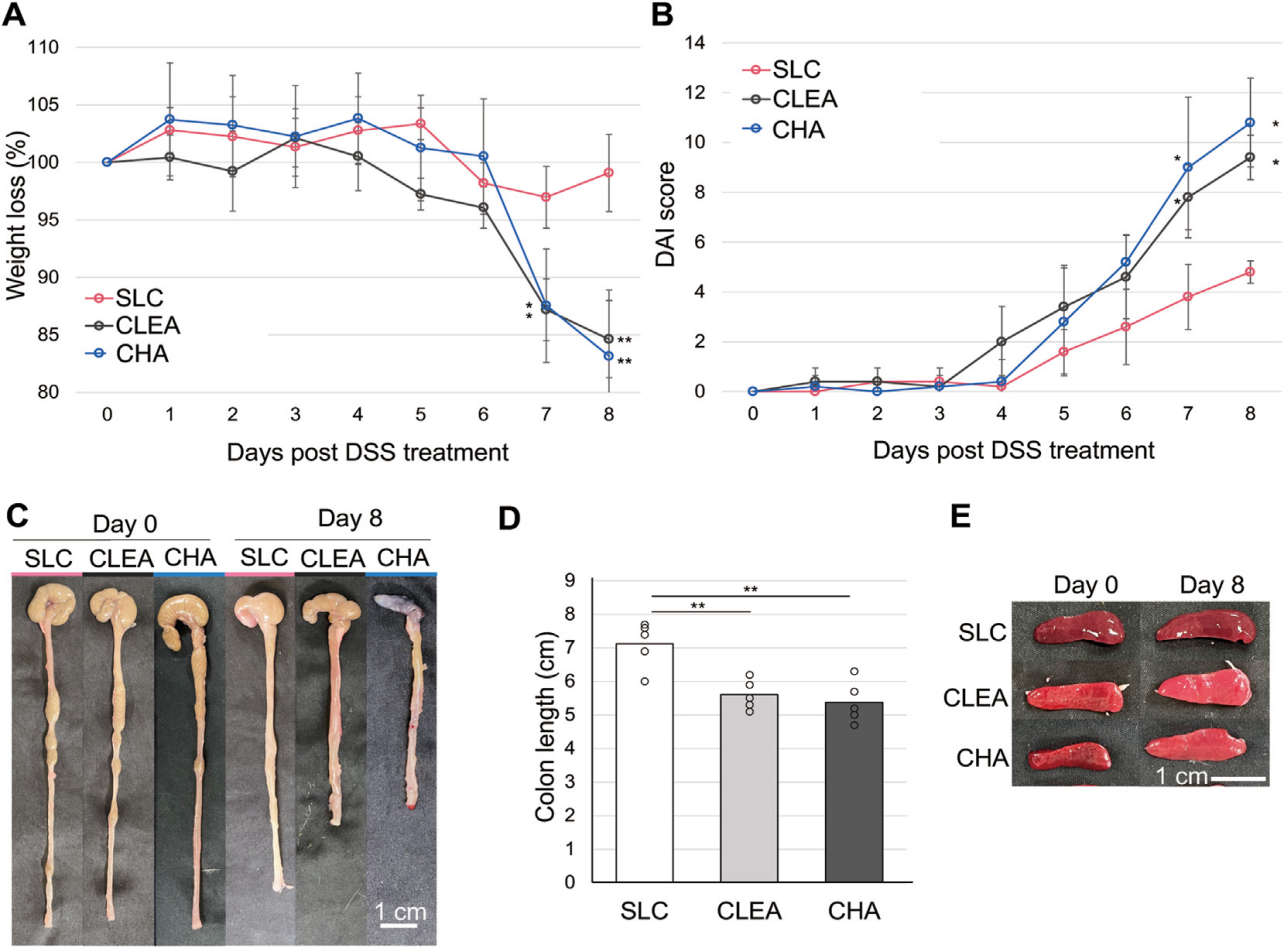
Clinical symptoms of colitis are highly variable among mice from different vendors. Mice from 3 commercial vendors, SLC, CLEA, and Charles River (CHA), were treated with 4% DSS for 8 days. Mice were evaluated daily, and weight loss and disease activity index scores were recorded. (A) Body weight changes, (n = 5). (B) DAI score, a score from 0 to 12. A higher number indicates more severe colitis (n = 5). (C) Gross images of the colon on days 0 (pre-DSS) and 8 (post-DSS). (D) Colon length was measured on day 8. (E) Gross images of the spleen on days 0 and 8. ∗*P* < .05, ∗∗*P* < .01.

We compared female six-to seven-week-old BALB/C wild-type mice obtained from 3 vendors: SLC, CLEA, and Charles River. Acute colitis was induced by the administration of 4% (w/v) DSS in drinking water for 8 days. To investigate whether colitis induction by DSS was comparable among mice from different vendors, weight loss, rectal bleeding, stool consistency, colon shortening, and spleen enlargement were observed as clinical features of disease. Regarding weight loss, the body weights of mice from all vendors slightly increased during the first few days of the experiment. The weight of CLEA mice and Charles River mice gradually began to decrease afterward, and the body weights on day 8 were 14.4 ± 9.7 and 16.8 ± 5.7% below the initial weight, respectively (Figure 1A). SLC mice showed little weight loss, and the body weight on day 8 was only 1.5 ± 4.1% below the initial weight. The level of weight loss in CLEA mice and Charles River mice significantly differed from that in SLC mice on day 7 (*P* = .04 and .03, respectively) and day 8 (*P* = 8.0 × 10^-3^and 4.0 × 10^-3^, respectively). Similarly, the DAI score, which comprises the degrees of weight loss and intestinal bleeding, was significantly higher (indicating more severe colitis) in CLEA and Charles River mice than in SLC mice on day 7 (*P* = .04 and .02, respectively) and day 8 (*P* = .02 and .02, respectively) (Figure 1B). The DAI score ranges from 0 to 12 (total score). Moreover, SLC mice did not exhibit considerable colon shortening (Figure 1C and D) or spleen enlargement (Figure 1E), unlike CLEA or Charles River mice. There was a trend of more colitis symptoms in Charles River mice than in CLEA mice, although significant differences in these symptoms of colitis were not observed.

### The Gut Microbial Compositions of BALB/c Mice Largely Varied Among Mice From Different Vendors

**Figure 2:**
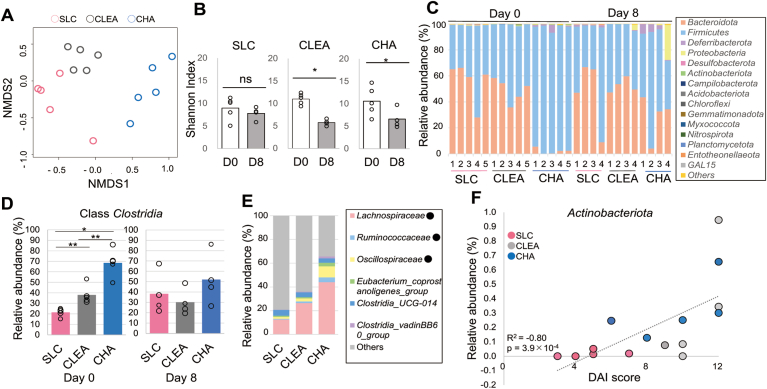
The gut microbiota in mice from 3 vendors assessed using 16S rRNA sequencing. The colorectal microbial composition in SLC, CLEA, and Charles River (CHA) mice treated with 4% DSS for 8 days was assessed using 16S rRNA amplicon sequencing. n = 4–5. (A) A nonmetric multidimensional scaling analysis identified a clear difference among mice vendor SLC (pink), CLEA (grey), and CHA (blue) before DSS treatment. (B) Dot plots show species-level microbial diversity measured by the Shannon diversity index. (C) Relative abundance of bacterial phyla presents in faeces on days 0 (pre-DSS) and 8 (post-DSS). (D) Relative abundance of class *Clostridia* comprising the phylum Firmicutes in faeces on days 0 (pre-DSS) and 8 (post-DSS). (E) Relative abundance of the bacterial family comprising the class *Clostridia* that was ≥ 1% abundant in at least one group of mice before DSS treatment. • Significantly different among vendors indicates. (F) Relationship between DAI score and relative abundance of phyla *Actinobacteriota* in mice from vendor SLC (pink) CLEA (grey), and CHA (blue) before DSS treatment (R^2^ = 0.83). ∗*P* < .05, ∗∗*P* < .01.

**Figure 3:**
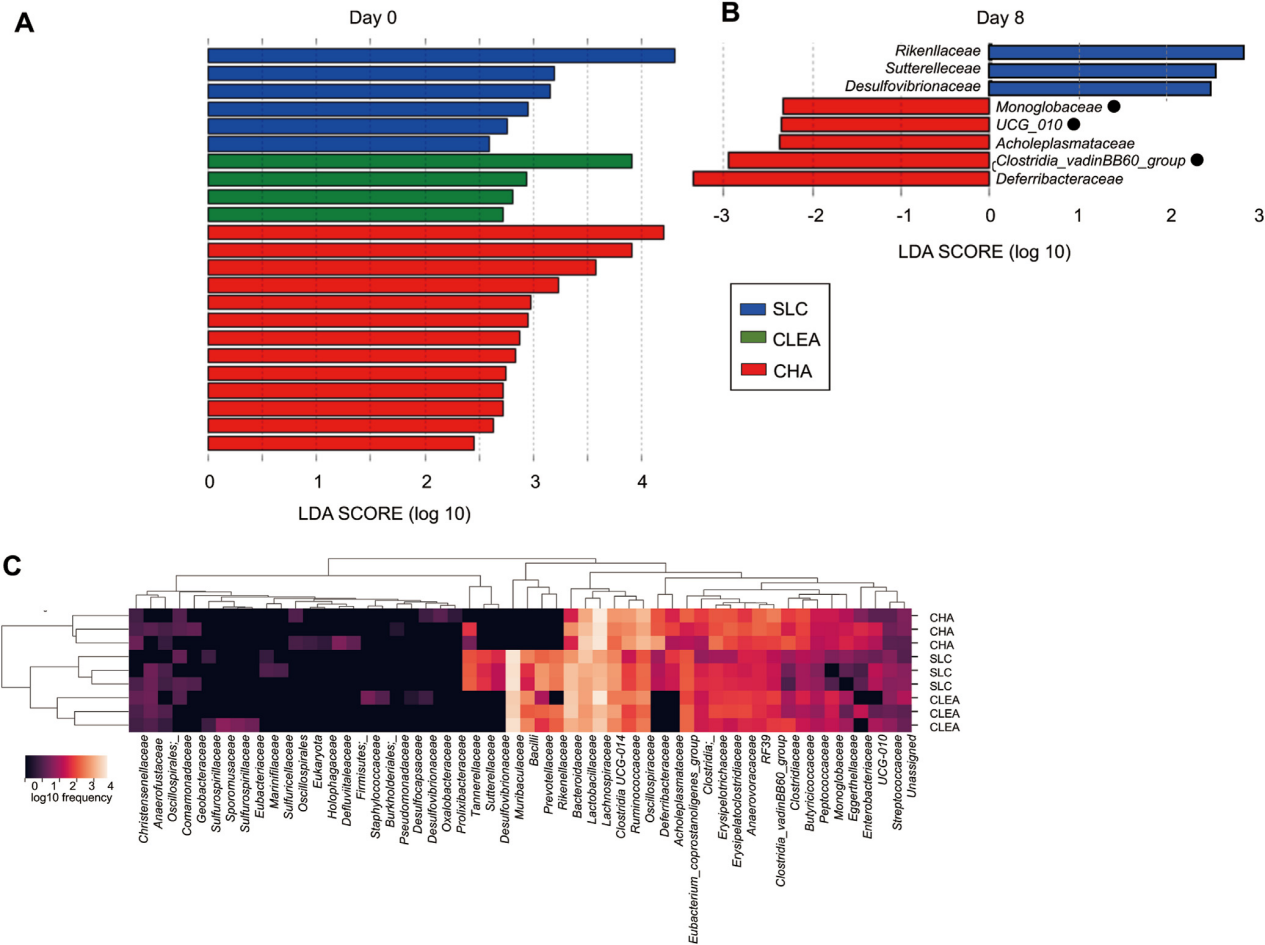
Differences in the microbiota of mice from the 3 vendors at the family level. Differences in microbiota taxa at the family level among mice from the 3 vendors were calculated by LDA effect size (LEfSe) on day 0 (A) and day 8 (B) (n = 4–5). • Bacterial taxa comprising the class *Clostridia*. (C) Heatmap showing bacterial family frequency distribution across mice from the 3 vendors before DSS treatment (n = 3).

**Figure 4:**
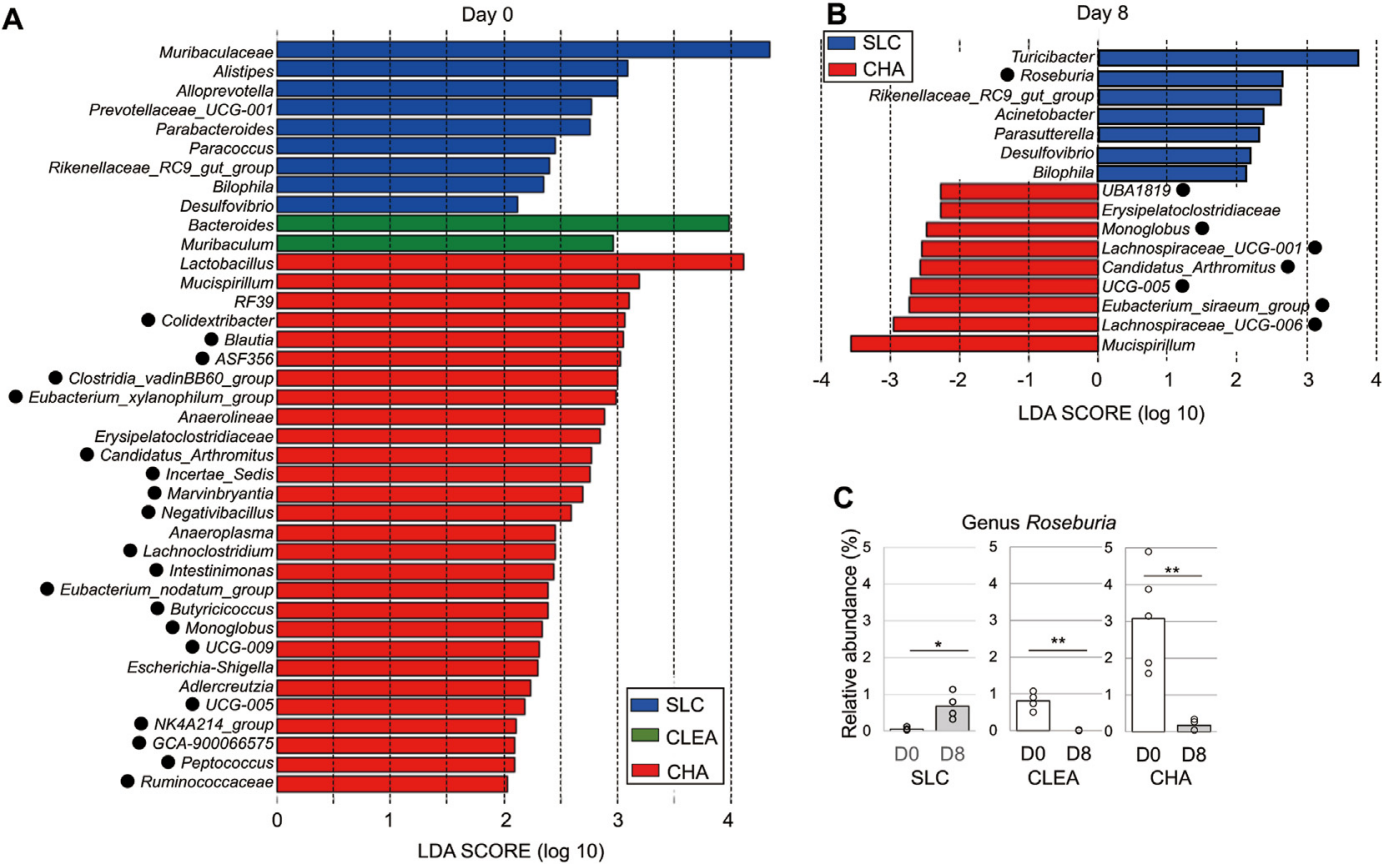
Differences in the microbiota of mice from the 3 vendors at the genus level. Differences in microbiota taxa at the genus level among mice from the 3 vendors were calculated by LDA effect size (LEfSe) on day 0 (A) and day 8 (B). • Bacterial taxa comprising the class *Clostridia*. (C) Relative abundance of the butyrate-producing genus *Roseburia* of mice before and after DSS treatment.

We compared the gut bacterial composition of mice from SLC, CLEA, and Charles River using 16S rRNA sequencing. Nonmetric multidimensional scaling ordination using Horn-Morisita dissimilarities based on community membership indicated a clear separation of the microbiota among mice from the different vendors before DSS treatment (Figure 2A). Consistent with previous studies, a significant decrease in microbial diversity, which is a characteristic of dysbiosis, was observed in CLEA and Charles River mice, whereas SLC mice did not show diversity changes after DSS administration (Figure 2B). Microbial diversity was not significantly different among vendors either before or after DSS administration. Fifteen phyla were identified in total, as shown in Figure 2C. Contrary to our expectations, Charles River mice, but not SLC mice, were distinct from mice from other vendors before DSS treatment. In other words, SLC mice and CLEA mice were similar despite differences in disease severity. Notably, although *Bacteroidetes* and *Firmicutes* are 2 main phyla of the gut microbiota, the phylum *Firmicutes* was the most dominant in Charles River mice before DSS treatment, comprising up to 95%; hence, a low abundance of *Bacteroidetes* (2.2%) was observed. The phylum *Firmicutes* consists of the class *Bacilli* and class *Clostridia* (Figure A1), and the levels of *Clostridia* in mice before DSS treatment was significantly lower in mice from SLC compared to CLEA and Charles River (Figure 2D), suggesting that the high *Firmicutes* abundance in Charles River mice was mainly due to the class *Clostridia*. The levels of *Clostridia* in mice after DSS treatment was not significantly different among vendors. Further investigation of the taxa comprising the class *Clostridia* was performed, and the levels of abundant families that were ≥ 1% abundant in at least one group are shown in Figure 2E. A decrease in the levels of SCFA-producing *Clostridium* cluster IV (*Ruminococcaceae* and *Clostridia UCG-014*) and XIVa (*Lachnospiraceae*) is often associated with gut dysbiosis.^30^ The levels of *Lachnospiraceae* and *Rumicococcaceae* in mice before DSS treatment were significantly lower in mice from SLC compared to CLEA and Charles River. The level of *Oscillospiraceae* in mice before DSS treatment was significantly lower in mice from SLC compared to Charles River. In addition to the presence of 2 main phyla, *Firmicutes* and *Bacteroidetes*, the analysis of the microbiota before DSS treatment showed a very strong association between the DAI inflammation score and *Actinobacteriota* proportion (R^2^ = 0.80), although the abundance of this microbe was quite low (Figure 2F and Figure A2). To distinguish the bacterial taxa that are commonly found in mice from each vendor, differences in microbial taxa at the family level among vendors were calculated by LEfSe as well as a heatmap (Figure 3 and Figure A3). Eight taxa out of 13 that were significantly abundant in Charles River mice before DSS treatment were from the class *Clostridia* except *Lactobacillaceae*, *RF39, Deferribacteraceae*, *Erysipelatoclostridiaceae, Acholeplasmataceae*, and *Eggerthellaceae.* The family *Eggerthellaceae* is a member of phylum *Actinobacteriota,* and all *Actinobacteriota* bacteria found in this study belonged to the *Eggerthellaceae.* Contrary to the mice before DSS treatment, taxa comprising the class *Clostridia* were significantly lower levels in Charles Rimer mice after DSS treatment. Members of the family *Desulfovibrionaceae* and *Rikenllaceae* were significantly abundant in SLC mice both before and after DSS treatment. More detailed composition at genus level by LEfSe analysis indicated similar tendency (Figure 4A and B). Members of the genus *Desulfovibrio* and *Bilophila,* both comprising family *Desulfovibrionaceae,* were significantly abundant in SLC mice both before and after DSS treatment. Twenty taxa out of 28 that were significantly abundant in Charles River mice before DSS treatment were from the class *Clostridia* and there were no abundant taxa in mice after DSS treatment. Furthermore, genus *Roseburia* is an only taxon comprising the class *Clastridia* in SLC mice after DSS treatment (Figure 4B). Among the SCFAs producing bacteria, *Roseburia intestinalis* (a member of family *Lachnospiraceae*) and *Faecalibacterium prausnitzii* (a member of family *Oscillospiraceae*) are the primary butyrate producers in the human gut.^31^ The mean abundance of the genus *Roseburia* was higher in Charles River and CLEA mice than in SLC mice before DSS treatment (3.1%, 0.81%, and 0.05%, respectively), and the abundance decreased in Charles River and CLEA mice after DSS treatment, whereas that in SLC mice increased after DSS treatment (0.17, 6.4 × 10^-3^, and 0.68%, respectively) (Figure 4C). The genus *Faecalibacterium* was not identified in all samples.

### Commensal Intestinal Bacteria From Mice With Severe Colitis Influence the Severity of Disease in Mice With Milder Colitis

**Figure 5:**
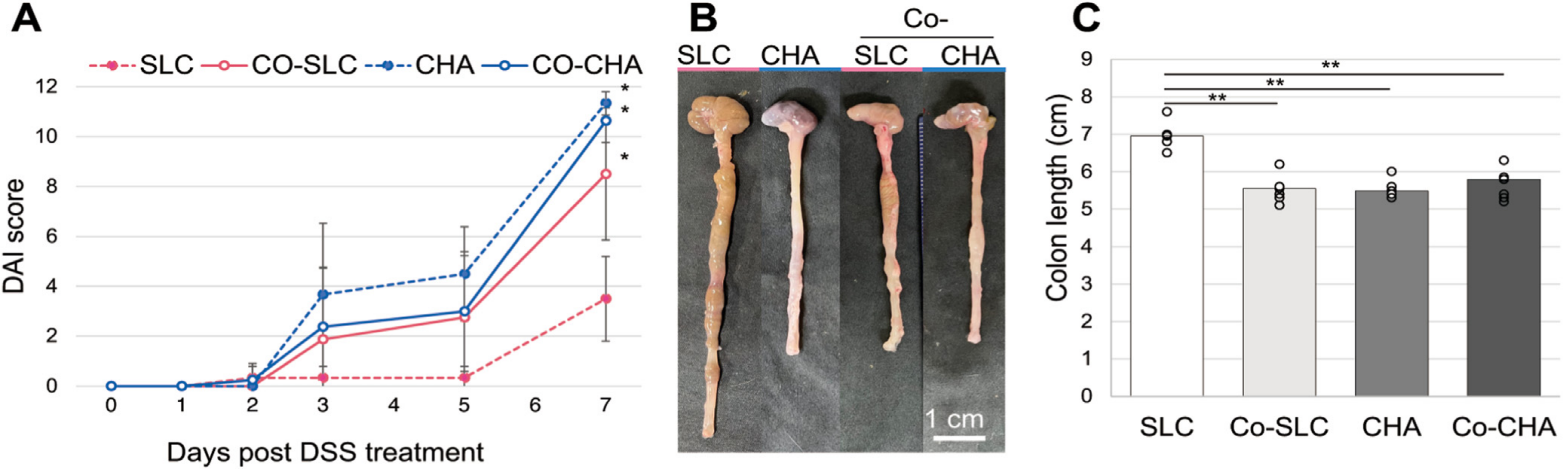
SLC mice cohoused with Charles River mice developed DSS-induced colitis. SLC mice (CO-SLC) and Charles River mice (CO-CHA) were cohoused for 4 weeks followed by 7 days of DSS administration (n = 8). SLC mice (SLC) or Charles River mice (CHA) that were kept in separate cages were used as controls (n = 6). Two independent experiments with identical results were combined. (A) DAI score. (B and C) Colon length was measured on day 7. ∗*P* < .05, ∗∗*P* < .01.

**Figure 6:**
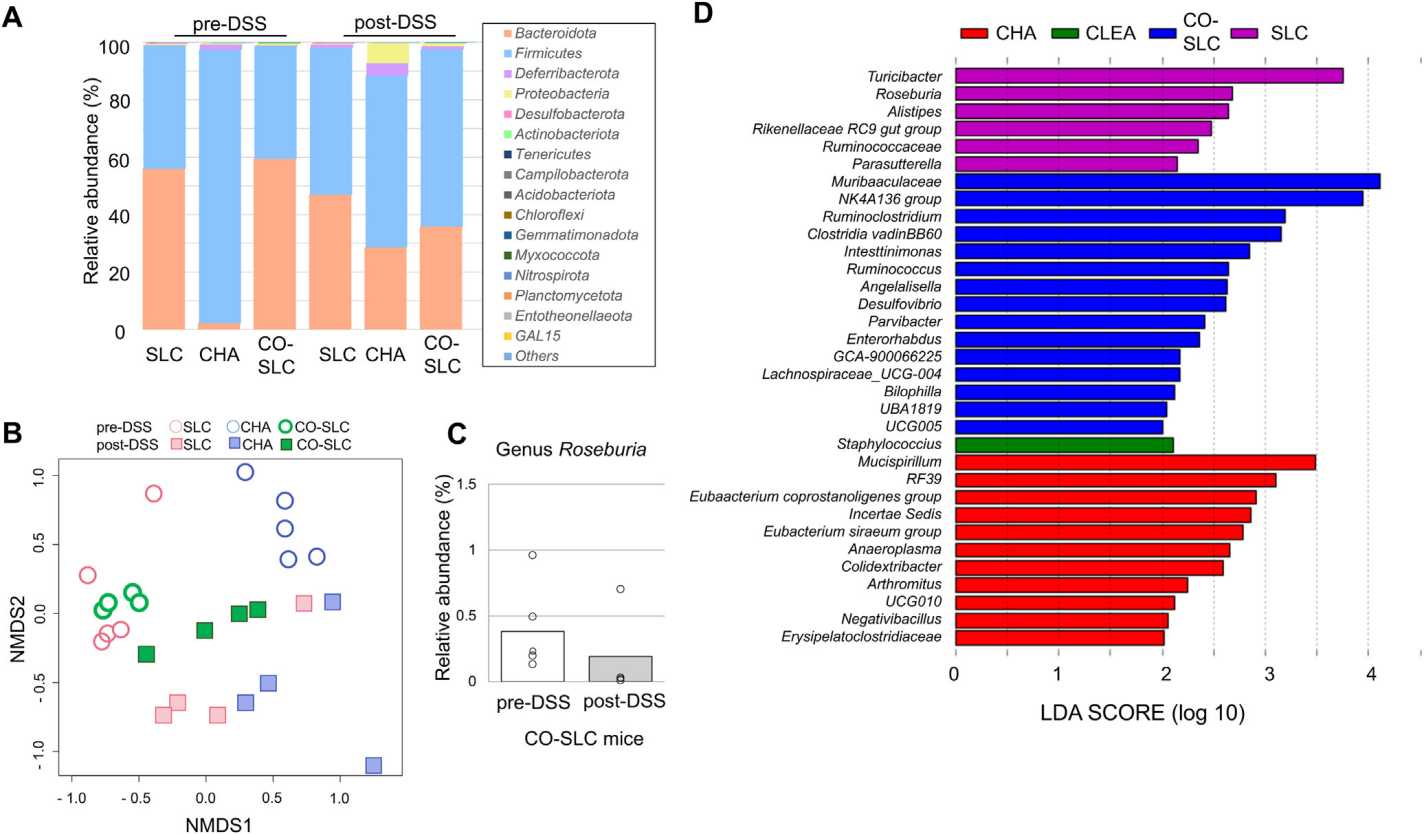
Comparison of gut microbiota from SLC mice cohoused with Charles River mice to solo housed mice from 3 vendors. The gut microbial composition in cohoused SLC mice (CO-SLC) was assessed using 16S rRNA amplicon sequencing and compared those from solo housed 3 vendors mice, SLC, CLEA, and Charles River (CHA). n = 4–5. (A) Relative abundance of bacterial phyla presents in faeces before and after DSS treatment. (B) A nonmetric multidimensional scaling analysis of gut microbial compositions among mice vendor CO-SLC (green), SLC (pink), and CHA (blue) before (circle) and after (square) DSS treatment. (C) Relative abundance of the butyrate-producing genus *Roseburia* of CO-SLC mice before and after DSS treatment. (D) Differences in microbiota taxa at the genus level among mice from the 3 vendors as well as CO-SLC mice were calculated by LDA effect size (LEfSe) after DSS treatment.

Our gut microbiota analysis suggested that specific colonic microbes may induce colitis susceptibility. We next aimed to address the possibility that disease resistance to colitis in SLC mice might have been due to genetic variants among BALB/c mice substrains from the different vendors. Therefore, we cohoused SLC mice (mildest colitis symptoms) with Charles River mice (severest colitis symptoms) to allow horizontal bacterial transmission. SLC mice that were cohoused with Charles River mice for 4 weeks showed statistically higher DAI scores than SLC mice that were kept in separate cages (day 7, *P* = .03), suggesting that the gut microbiota, rather than the presence of genetic variants, is a dominant factor in the variable response to DSS between these 2 mouse substrains (Figure 5A). Regarding Charles River mice, although both Charles River mice housed separately and cohoused mice showed significantly higher DAI values than SLC mice (day 7, *P* = .03 and .01, respectively), no change was observed due to cohousing with SLC mice. Similarly, more colon shortening was observed in cohoused SLC mice, separately housed Charles River mice, and cohoused Charles River mice than in separately housed SLC mice (*P* = 1.6 × 10^-4^, 2.0 × 10^-3^, and 1.7 × 10^-3^, respectively) (Figure 5B and C). We further analyzed the gut microbiota of cohoused SLC mice using 16S rRNA sequencing and compared bacterial compositions to those from solo housed SLC, CLEA, and Charles River mice. After 4 weeks of cohousing, SLC mice cohoused with Charles River mice maintained the *Firmicutes/Bacteroidota* balance and the bacterial compositions were generally similar to solo housing SLC (Figure 6A and B). Regarding the butyrate-producing genus *Roseburia*, the abundance of *Roseburia* in cohoused SLC mice was 0.38% after cohousing and decreased to 0.19% with DSS challenges. The decrease in *Roseburia* in cohoused SLC mice indicates loss of persistence of *Roseburia*, unlike disease-resistant solo housed SLC mice (Figures 4C and 6C). Furthermore, we performed LEfSe analysis to compare disease-resistant SLC mice with disease-susceptible Charles River, CLEA, and cohoused SLC mice following DSS treatment. The 6 genera were abundant in disease-resistant SLC mice, *Turicibater, Roseburia, Alistipes, Rikenellaceae RC9 gut group*, *Ruminococcaceae*, and *Parasutterella* (Figure 6D).

## Discussion

The DSS-induced colitis model is widely used because it can be established quickly and is simple.^24^ Here, we found clear differences in susceptibility to DSS-induced colitis among Japanese laboratory mice from 3 vendors. BALB/C mice purchased from SLC showed lower disease symptoms than mice from the other 2 vendors. The gut microbiota is known as an indispensable factor in gut inflammation.^32^ To quantify the connection between disease severity and the gut microbiota, the faeces of mice from 3 vendors collected before and after DSS treatment were compared using 16S rRNA sequencing, revealing that each group of mice from the different vendors harboured a distinct gut microbiota. Moreover, the cohousing data from the present study further show that the severity of DSS-induced colitis was mainly influenced by the gut microbiota.

The variability in DSS-induced colitis among individual mice from the same inbred strain has been documented in a large-scale animal experiment using genetically identical laboratory mice from a single animal facility.^33^ The researchers reported that the presence of specific gut bacteria was mainly responsible for the variable experimental outcomes in the DSS model. In humans, a large clinical cohort study examined genetic-microbial associations in healthy people who had different ancestral backgrounds but shared a relatively similar environment. The study showed that host genetics or ancestral backgrounds have a minor role in determining the gut microbiome; rather, the microbiota is shaped predominantly by lifestyle and is similar among individuals who share a relatively homogenous environment.^34^ IBD patients show a distinct distribution of certain bacterial taxa; IBD is accompanied by a decreased abundance of *Bacteroidetes, Firmicutes, Clostridia, Lactobacillus,* and *Ruminococcaceae* and an increased abundance of *Gammaproteobacteria* and *Enterobacteriaceae*.^35^^,^^36^ In particular, the *Firmicutes/Bacteroidetes* ratio is widely accepted to have vast influences on the maintenance of gut homeostasis, and an imbalance in these taxa can lead to various pathologies.^32^ Related to these epidemiological studies, our study revealed a higher percentage of *Firmicutes* in Charles River mice, and these bacteria were classified mainly into the class *Clostridia*. Among the *Clostridia*, members of *Clostridium* cluster IV (*Ruminococcaceae* and *Clostridia UCG-014*) and XIVa (*Lachnospiraceae*) are beneficial microbiota that produce SCFAs, including butyrate.^37^, ^38^, ^39^ In particular, *Roseburia intestinalis* is one of the primary butyrate producers in the human gut.^31^ The genus *Roseburia* consists of obligate gram-positive anaerobic bacteria, all of which are known to be SCFA producers.^40^ We reported that the abundance of the genus *Roseburia* increased precipitously in SLC mice after DSS treatment, whereas cohoused SLC mice as well as mice from other 2 vendors decreased the abundance of *Roseburia* along with DSS treatment. This implies that the persistence of *Roseburia* correlates with decreased susceptibility to disease in this setting. In addition to *Roseburia,* 5 genera were more abundant in disease-resistant SLC mice than in cohoused SLC mice and mice from other 2 vendors shown in Figure 6D. Some members of genera *Turicibater, Alistipes,* and *Ruminococcaceae* were also known as SCFA-producers.^41^ Similarly, in a previous study that compared the gut microbiota of mice from 2 vendors (substrains) in the United States, colonization of the gut by colitis-resistant *Candidatus arthromitus* (a segmented filamentous bacterium and member of the family *Clostridiaceae*) was found in Taconic mice.^26^ The distribution of disease-resistant bacteria may not be ubiquitous but may have similar characteristics among different mice.

As previously discussed, we showed that few colitis symptoms were observed in SLC mice in our study. Although SLC is a major animal manufacturer, there have been few reports of studies using mice from SLC in the field of DSS-induced colitis research. One report using male C57BL/6 mice from SLC described body weight loss as well as a low DAI score, which supports our data.^42^ C57BL/6, BALB/c, and C3H/HeJ strains are known to be genetically susceptible to DSS-induced colitis.^43^ To our knowledge, this is the first report to describe a disease-resistant BALB/c substrain with lower disease susceptibility, and we showed that the susceptibility mainly relies on the gut microbiota. While our gut microbiota analysis using 16S rRNA analysis had limitations in the accurate characterization of species, the levels of the family *Muribaculaceae,* which was statistically abundant in SLC mice, have been reported to be negatively correlated with DSS-induced inflammation and further preserved in DSS-resistant *Dusp*6 knockout mice.^44^, ^45^, ^46^ Considering these findings, members of the family *Muribaculaceae* could also be studied in the future as potential protective bacteria against disease.

In summary, a mouse substrain that was resistant to DSS-induced colitis was observed, and the severity of DSS-induced colitis was mainly influenced by the gut microbiota. DSS-induced colitis is one of the central preclinical models used in the gastrointestinal field. When studying disease susceptibility in laboratory mice, the mouse vendor and/or bleeding conditions that influence the gut commensal microbiota may be the reason for variable outcomes.

## Supplementary Materials

Figures A1–A3
